# Vertical implantable collamer lens as a novel method to increase rotational stability

**DOI:** 10.1371/journal.pone.0308830

**Published:** 2024-08-19

**Authors:** Yongwoo Lee, Sang Beom Han, Gerd U. Auffarth, Hyeck-Soo Son, Ramin Khoramnia, Chul Young Choi, Kun Moon, Sang Il An, Je Myung Lee, Jong Ho Lee

**Affiliations:** 1 Department of Ophthalmology, Kangwon National University Hospital, Kangwon National University School of Medicine, Chuncheon, Republic of Korea; 2 Saevit Eye Hospital, Goyang, Republic of Korea; 3 The David J. Apple International Laboratory for Ocular Pathology and International Vision Correction Research Centre (IVCRC), Department of Ophthalmology, University of Heidelberg, Heidelberg, Germany; 4 Department of Ophthalmology, Kangbuk Samsung Hospital, Sungkyunkwan University School of Medicine, Seoul, Republic of Korea; 5 Seoulbalgeunsesang Eye Clinic, Seoul, Republic of Korea; Keio University School of Medicine, JAPAN

## Abstract

**Purpose:**

We investigated the vertical implantation of a toric implantable collamer lens (ICL) and compared the rotational stability with that of horizontal implantation.

**Methods:**

This matched comparative study retrospectively reviewed and analyzed data from patients who underwent ICL implantation from 2003–2022 by 1:1 matching vertical and horizontal (V and H toric groups, respectively) implantation patients according to preoperative astigmatism, spherical equivalent, sulcus-to-sulcus, anterior chamber depth, and ICL size. Visual acuity, manifest refraction, vaulting, and rotation were measured 3 months postoperatively.

**Results:**

We included 646 eyes (323 each in the V and H toric groups). No statistically significant difference was observed between groups in postoperative visual acuity, refractive error, and astigmatism. Vaulting was lower in the V toric group. (P < 0.001). The mean lens rotation in the V toric group was less than that in the H toric group (1.11 ± 2.84° versus 3.02 ± 10.34°, P = 0.001). The proportion of eyes in the V and H toric groups showing ≥10° of rotation was 2.5% (8 eyes) and 6.5% (21 eyes), respectively (P = 0.014). Despite repositioning from rotation, three (0.9%) and eight (2.5%) eyes required removal owing to lens re-rotation in the V and H toric groups, respectively.

**Conclusion:**

Toric ICL vertical implantation showed good rotational stability, and appropriate visual acuity correction results with relatively low vaulting. This procedure therefore presents an effective novel method that could replace horizontal toric ICL implantation.

## Introduction

An implantable collamer lens (ICL) can be inserted in the posterior chamber to correct myopia in patients with high myopia or thin corneas for whom corneal resection is insufficient. ICLs play an important role in vision correction, with advantages in that relatively high myopia can be corrected and complications such as corneal opacity or ectasia can be avoided [1, 2]. However, due to the nature of the lens that must be inserted into the sulcus, high vaulting, a narrow anterior chamber angle, or decreased number of corneal endothelial cells can cause serious complications. Moreover, the lower the vaulting, the greater the risk of cataracts [3]. Hence, suitable vaulting is generally recommended between 250 and 750 um [4].

However, when relatively high vaulting occurs in clinical practice, endothelial cell reduction, iris chafing or peripheral anterior synechiae cause irreversible damage; therefore, the lens size should be considered to ensure the lowest possible vaulting. The ICL is generally inserted horizontally while considering the distance between the sulcus, based on the white-to-white (WTW) distance, as the manufacturer recommends. Then, if the vaulting is higher than expected, surgery must be performed to replace the lens [4–6]

**Fig 1:**
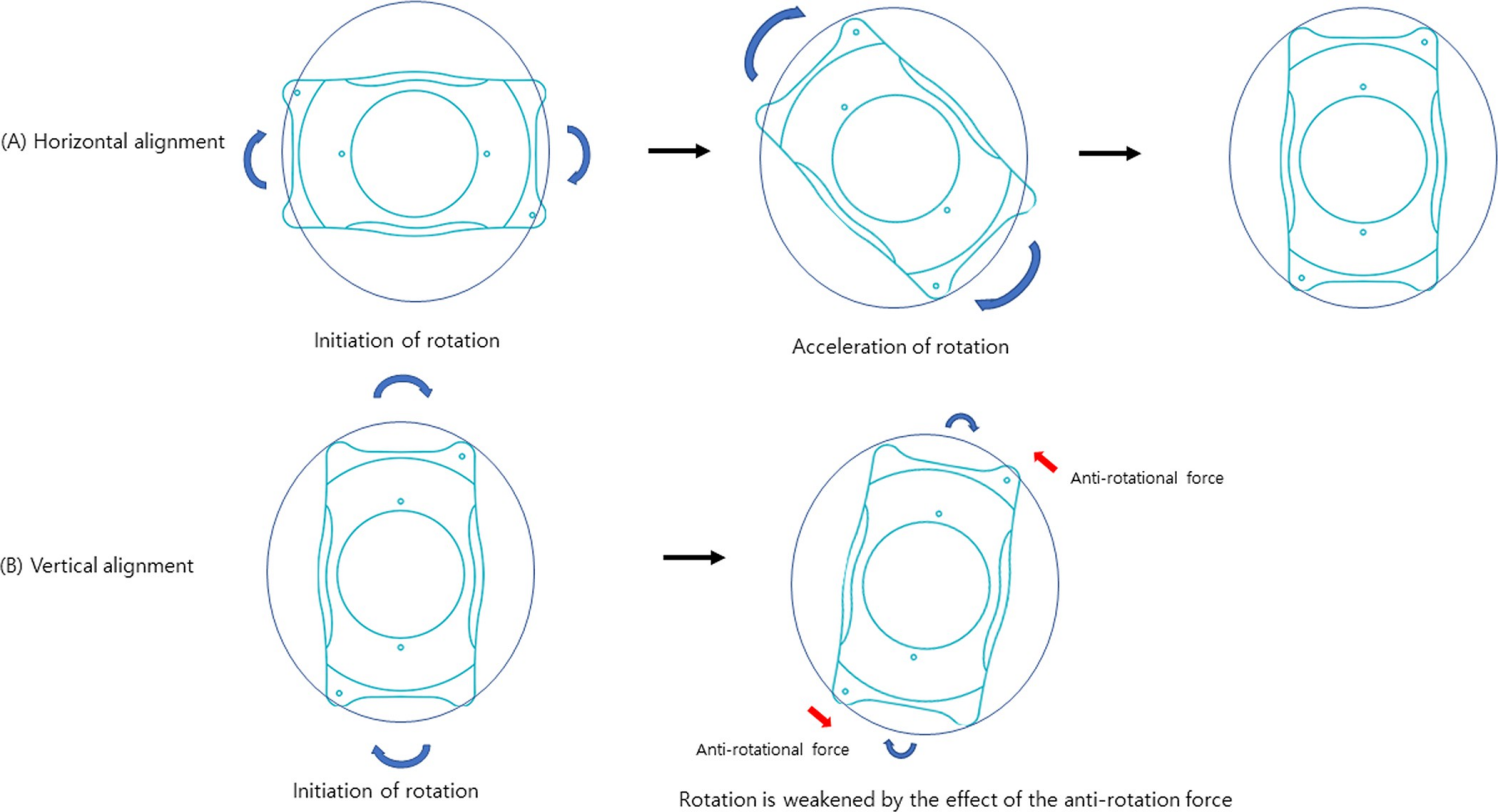
Diagram of vertical phakic intraocular lens. Schematic illustration of how a horizontal implantable collamer lens (ICL) implantation is more prone to higher degree of rotation in case of a rotation compared to a vertical ICL insertion which tends to rotate less thanks to an anatomical anti-rotational force.

Since 2011, the AQUA ICL (Visian ICL with Central FLOW technology, V4C; STAAR surgical company, Monrovia, CA, USA), which has a hole in the center of the lens, has been released, eliminating the need for upper peripheral iridotomy. This design has allowed a potential reduction of long-term ICL complications such as iris block or cataracts [4]. Considering that the distance between the vertical sulcus is generally wider than that of the horizontal sulcus [7], if the vaulting of the horizontally inserted ICL is too high, a relative reduction can be achieved by rotating it vertically [8]. However, this method is only possible using a non-toric lens. Moreover, with toric ICLs, the axis of astigmatism is distorted when rotated. Since the vertical sulcus distance is longer, it rotates more than 10° when the toric lens rotates; therefore, many patients require lens removal and astigmatism correction using laser-assisted in situ keratomileusis after the non-toric lens is inserted (Fig 1).

Since 2019, we have inserted toric ICLs manufactured vertically for vertical toric ICL implantation to maintain rotational stability and low vaulting. Therefore, we aimed to perform a comparative study of the clinical results and rotation of hundreds of vertical ICL cases with those of horizontal toric ICL cases.

## Methods

This study was conducted in accordance with the Declaration of Helsinki and was approved by the Institutional Review Board of Kangwon National University Hospital (No. 2022-12-016). The board waived the need for written informed consent due to the retrospective nature of the study. We retrospectively analyzed the medical records of patients who underwent ICL with central hole implantation at Seoul Balgeunsesang Eye Clinic from Jan. 2011 to Dec. 2022. Data for the study were accessed from January 4, 2023. The data was collected in such a way that individual information could not be identified. Rotation was compared using 1:1 matching by astigmatism (within 0.25 D), sulcus-to-sulcus (STS) distance (within 0.1 mm), anterior chamber depth (ACD; within 0.1 mm), and ICL size (12.1, 12.6, 13.2, or 13.7 mm) between the eyes that received horizontal toric ICL (H toric group) and those that received vertical toric ICL (V toric group). Patients who had an ICL without a central hole were excluded from matching. To minimize the influence on rotation, cases in which there was a difference between the astigmatic axis of the patient and that of the lens during lens manufacturing were excluded.

### Preoperative examination

All patients underwent a complete preoperative ophthalmologic examination, including corrected visual acuity, manifest refraction (MR), slit lamp examination, intraocular pressure (IOP), specular microscopy, and mydriatic fundus examinations. In all patients, the OCULUS Pentacam^®^ (OCULUS Optikgeräte GmbH, Wetzler, Germany) was used to measure the ACD and WTW distance, while the STS distance was measured horizontally and vertically using an ultrasound biomicroscope (VuMax™, Sonomed Escalon, New Hyde Park, NY, USA), in all patients.

### Surgical procedure

To facilitate lens insertion, the size and power of the ICL were determined by inputting the MR, keratometry, WTW distance, corneal thickness, and ACD using the program provided by the manufacturer (STAAR Surgical AG, Nidau, Switzerland). For the ICL power, we selected the myopia power and astigmatism power closest to 0 D provided through the manufacturer’s program. For vertical insertion, the axis of astigmatism was changed by 90 degrees and entered into the manufacturer’s program, designed for horizontal production. In order to reduce the vaulting as much as possible, a smaller lens was selected in cases of ambiguous sizes. The operations were performed by three experienced surgeons (YWL, SIA, JML).

With the patient in an upright seated position, marks were accurately made at the 3, 9, and 12-o’clock positions using a slit beam [9]. Mydriasis was achieved by instilling 0.5% tropicamide/phenylephrine hydrochloride (Mydrin-P^®^, Santen Pharmaceutical Co., Ltd., Osaka, Japan) and anesthesia by instilling 0.5% proparacaine HCL (Alcaine^®^, Alcon Laboratories, Inc., Fort Worth, TX, USA). A 3-mm incision was made on the superior limbus using a diamond knife, and viscoelastic material (Provisc^®^, Alcon Laboratories, Inc.) was injected. The lens was inserted using an ICL injector system (the STAAR ICL injector system) horizontally or vertically on the marked axis. The lens was placed behind the iris using a manipulator to complete the operation, and the viscoelastic material was removed using a balanced salt solution (Alcon Laboratories, Inc.).

### Postoperative examination

**Fig 2:**
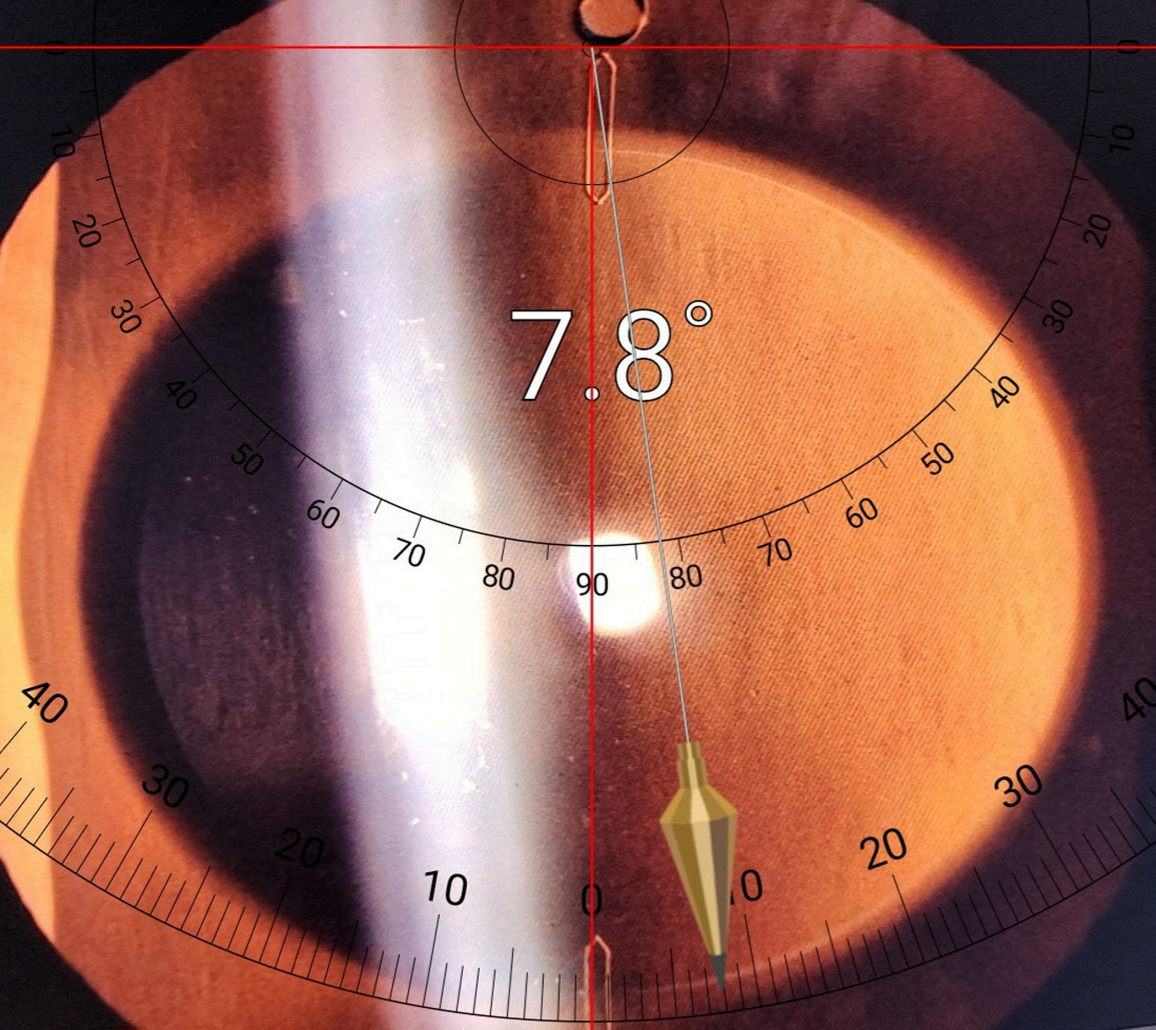
Rotation measurement of implantable collamer lens using smartphone protractor application and slit lamp image.

Follow-up was performed on the first day, week, and month postoperatively, the third month postoperatively, and annually thereafter. Three months after surgery, the uncorrected and corrected visual acuity, MR, IOP, lens rotation by dilation, and vaulting were measured using anterior optical coherence tomography (Cirrus 6000, Carl Zeiss Meditec AG, Jena, Germany). To eliminate the influence of the pupil on vaulting, vaulting was measured in all patients following pupil dilation [10]. To ensure accurate angle measurement, anterior segment images were obtained, and the rotation angle was measured by inserting the image into a smartphone protractor application (Smart Protractor, Smart Tools Co., Daegu, Korea; Fig 2). If the visual acuity deteriorated significantly owing to rotation, repositioning was performed once. In re-rotation after repositioning, the toric lens was removed, a non-toric ICL was inserted, and the astigmatism was subsequently corrected with laser ablation.

### Statistical analysis

The size of the study subjects was calculated using the g-power program (ver. 3.1.9.7, Heinrich-Heine-Universität Düsseldorf, Düsseldorf, Germany). For a power of 0.99, 296 cases were required (effect size 0.5, alpha-error: 0.05), and more than 300 cases were collected through a 1:1 match.

Statistical analysis was performed using SPSS version 25.0 (IBM, Armonk, NY, USA). We used the Kolmogorov-Smirnov test to verify the normal distribution of the data. An independent t-test was performed to compare numeric variables; the chi-square test was performed to compare categorical variables. P values less than 0.05 were considered statistically significant. To ensure statistical accuracy, only the right eye was included in the statistical analysis when both eyes met the criteria.

## Results

Of the 16,281 eyes, 1,850 had lenses inserted vertically (the rest, 14,431, had been inserted using the prior horizontal method). Six hundred forty-six eyes from 646 individuals were selected according to the matching criteria (323 eyes each in the V and H toric groups, respectively). Table 1 demonstrates the demographic and preoperative characteristics of both groups. The average participant age was 24.70 ± 4.89 and 24.82 ± 4.67 years in the V and H toric groups, respectively (p = 0.742). The spherical equivalent (SE) was –8.60 ± 1.91 and –8.55 ± 1.89D in the V and H toric groups, respectively (p = 0.770). Preoperative ophthalmologic measures such as logarithm of the minimum angle of resolution corrected distance visual acuity (logMAR CDVA), astigmatism, IOP, keratometry, WTW and STS distances, ACD, and corneal thickness showed no statistically significant differences between groups (Table 1).

**Table 1:** Demographics and preoperative ocular parameters of vertical and horizontal implantable collamer lens insertion group.

	V toric group (n = 323)	H toric group (n = 323)	P value
Age	24.70 ± 4.89 (20.00,42.00)	24.82 ± 4.67 (20.00, 42.00)	0.742 ^a^
Female	166/323 (51.4%)	162/323 (50.2%)	0.753 ^b^
MR			
SE (D)	–8.60 ± 1.91 (–14.88, –3.50)	–8.55 ± 1.89 (–14.63, –2.75)	0.770 ^a^
Cyl (D)	–2.73 ± 0.84 (–6.00, –1.00)	–2.71 ± 1.89 (–6.00, –1.00)	0.357 ^a^
IOP (mmHg)	16.53 ± 2.85 (10.00, 21.00)	16.47 ± 2.93 (9.00, 21.00)	0.796 ^a^
LogMAR CDVA	–0.05 ± 0.04 (–0.08, 0.00)	–0.06 ± 0.07 (–1.08, 0.00)	0.396 ^a^
flat K (D)	42.85 ± 2.12 (39.01, 46.25)	42.74 ± 2.17 (39.37, 46.25)	0.527 ^a^
steep K (D)	45.66 ± 1.39 (41.74, 50.49)	45.51 ±1.46 (40.87, 50.25)	0.167 ^a^
K astigmatism (D)	2.81 ± 1.92 (1.25, 6.50)	2.76 ± 1.88 (1.25, 6.50)	0.745 ^a^
WTW (mm)	11.62 ± 0.32 (10.70, 12.30)	11.62 ± 0.31 (10.70, 12.40)	0.822 ^a^
H-STS (mm)	11.32 ± 0.60 (10.70, 12.30)	11.33 ± 0.60 (10.80, 12.30)	0.854 ^a^
V-STS (mm)	11.60 ± 0.28 (11.0, 12.70)	11.61 ± 0.28 (11.00, 12.70)	0.981 ^a^
ACD (mm)	3.36 ± 0.71 (2.92, 3.85)	3.36 ± 0.70 (2.92, 3.84)	0.988 ^a^
CT (um)	531.32 ± 35.55 (446.00, 628.00)	531.07 ± 36.97 (436.00, 628.00)	0.931 ^a^

MR Manifest refraction; SE spherical equivalent; D diopters; Cyl cylinder; IOP intraocular pressure; LogMAR CDVA logarithm of minimal angle of resolution corrected distance visual acuity; K keratometry; WTW white to white; H-STS horizontal sulcus to sulcus; V-STS vertical sulcus to sulcus; ACD anterior chamber depth; CT corneal thickness

^a^Independent sample t-test

^b^Chi-square test

Values are shown as mean ± standard deviation (maximum, minimum)

Table 2 shows the postoperative outcomes. The logMAR uncorrected distance visual acuity at 3 months postoperatively was –0.03 ± 0.07 and –0.02 ± 0.17 in the V and H toric groups, respectively, with no statistically significant difference (p = 0.290). No significant difference was observed in logMAR CDVA (P = 0.162), SE (P = 0.468), and IOP (P = 0.595) between groups. The mean postoperative astigmatism was -0.58 ± 0.46 and -0.64 ± 0.44 in the V and H toric groups, respectively. Although not statistically significant, slightly better results were observed in the V toric group (P = 0.054). Vaulting was 462.24 ± 117.89 μm in the V toric group, which was lower than that in the H toric group (539.52 ± 154.00 μm; P < 0.001). The postoperative rotation of the ICL axis was 1.11 ± 2.84° in the V toric group, which was statistically significantly less than that in the H toric group (3.02 ± 10.34°; P = 0.001; Table 2)

**Table 2:** Postoperative outcomes, vaulting and rotation of vertical and horizontal implantable collamer lens insertion group.

	V toric group (n = 323)	H toric group (n = 323)	P value^a^
LogMAR UDVA	–0.03 ± 0.07 (–0.18, 0.30)	–0.02 ± 0.17 (–0.18, 0.30)	0.290
LogMAR CDVA	–0.07 ± 0.03 (–0.18, 0.5)	–0.06 ± 0.06 (–0.18, 0.15)	0.162
MR			
SE (D)	–0.12 ± 0.35 (–2.13, 0.75)	–0.14 ± 0.42 (–1.50, 1.50)	0.468
Astigmatism (D)	0.58 ± 0.46 (0.00, 4.25)	0.64 ± 0.44 (0.00, 2.00)	0.054
IOP (mmHg)	15.49 ± 2.85 (9.00, 21.00)	15.50 ± 2.80 (9.00, 20.00)	0.595
Vaulting (um)	462.24 ± 117.89 (145.80, 988.00)	539.52 ± 154.00 (159.00, 1270.00)	**<0.001**
Rotation (˚)	1.11 ± 2.84 (0.00, 26.00)	3.02 ± 10.34 (0.00, 90.00)	**0.001**

LogMAR UCVA logarithm of minimal angle of resolution uncorrected distance visual acuity; LogMAR CDVA logarithm of minimal angle of resolution corrected distance visual acuity; MR Manifest refraction; SE spherical equivalent; D diopters; Cyl cylinder; IOP intraocular pressure

^a^Independent sample t-test

Values are shown as mean ± standard deviation (maximum, minimum). Statistical significance is indicated in bold.

Overall, 147 eyes (45.5%) in the V toric group showed no postoperative lens rotation, which was statistically significantly higher compared to 101 eyes in the H toric group (31.3%) with no lens rotation (P <0.001; Table 3). The proportions of eyes with ≤1° (P < 0.001) and ≤3° (P = 0.032) of rotation were significantly higher in the V toric group. The rate of rotation of 7° or more was observed in 12 eyes (3.7%) in the V toric group, which was less than that observed in the H toric group (24 eyes [7.4%]) (P = 0.040). Furthermore, the number of eyes with ≥10° of lens rotation was 8 (2.5%) in the V toric group compared to 21 (6.5%) in the H toric group, and the statistical significance of the difference became clearer (P = 0.014). Rotation of ≥30° was observed in seven eyes in the H toric group (2.2%) and none in the V toric group (P = 0.008; Table 3)

**Table 3:** Comparison of number of eyes that showed postoperative rotation or that required reposition, re-rotation after repositioning, or lens exchange between the vertical and horizontal implantable collamer lens insertion groups (V toric group and H toric group).

	V toric (n = 323)	H toric (n = 323)	P value ^a^
No rotation	147 (45.5%)	101 (31.3%)	**<0.001**
Rotation ≤ 1°	291 (90.1%)	250 (77.4%)	**<0.001**
Rotation ≤ 3°	310 (96.0%)	297 (92.0%)	**0.032**
Rotation ≥ 7°	12 (3.7%)	24 (7.4%)	**0.040**
Rotation ≥ 10°	8 (2.5%)	21 (6.5%)	**0.014**
Rotation ≥ 30°	0 (0.0%)	7 (2.2%)	**0.008**
Need for reposition	16 (5.0%)	24 (7.4%)	0.146
Re-rotation after repositioning	8 (2.5%)	11 (3.4%)	0.485
Exchange due to re-rotation	3 (0.9%)	8 (2.5%)	**0.031**

^a^Chi-square test

Statistical significance is indicated in bold.

In the V toric group, the average vaulting of patients who required rotation was 430.94 ± 116.73 μm, which was lower than that of patients who did not require rotation (463.87 ± 116.73 μm), but this difference was not statistically significant (P = 0.277). In the H toric group, the average vaulting of patients who required rotation was 495.19 ± 234.71 μm, which did not show a significant difference compared to the average vaulting of patients who did not require rotation (485.58 ± 230.89 μm).

**Fig 3:**
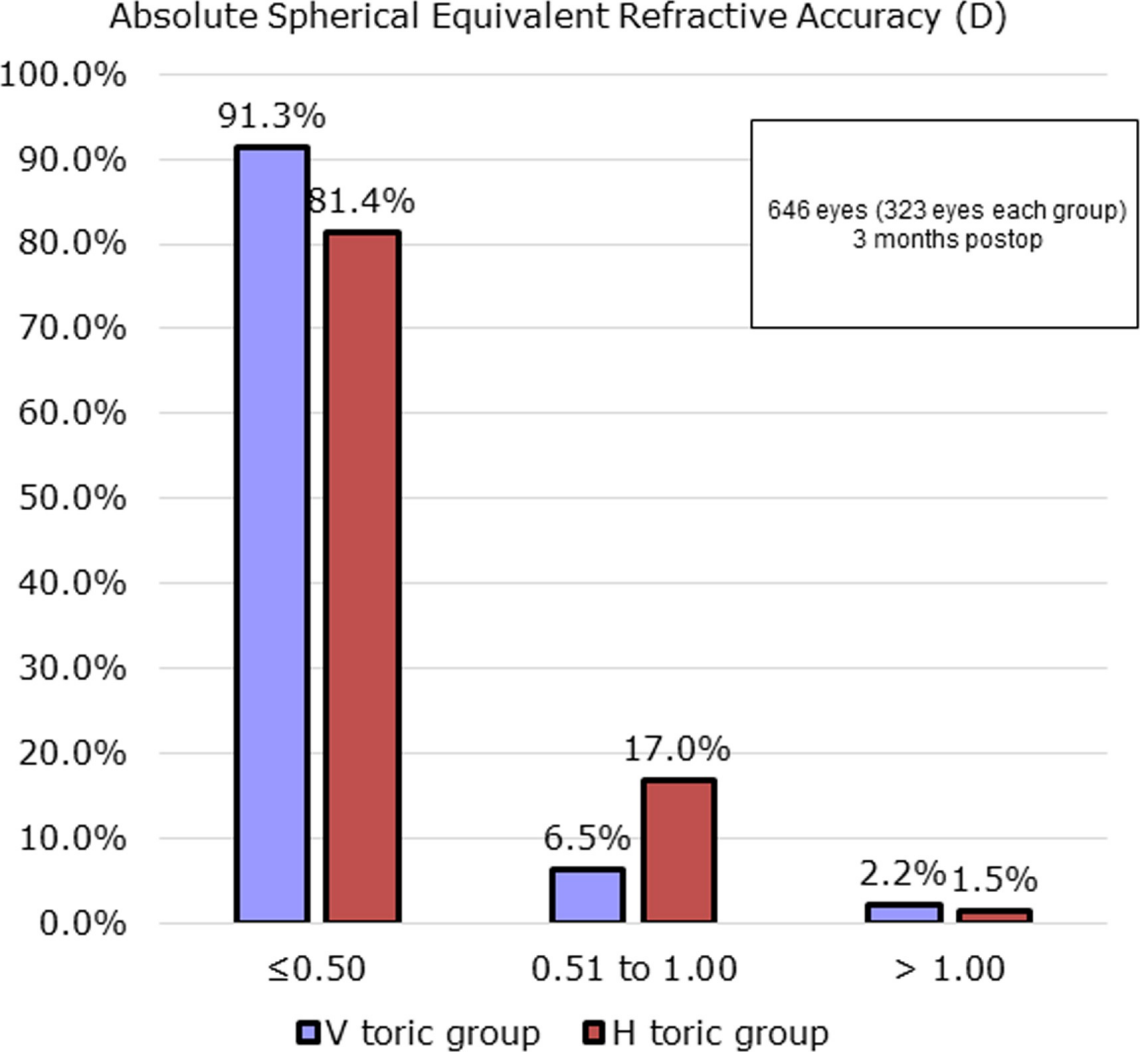
Spherical equivalent refractive accuracy of vertical and horizontal implantation groups measured at 3-months postoperatively. N = 323 in each group.

**Fig 4:**
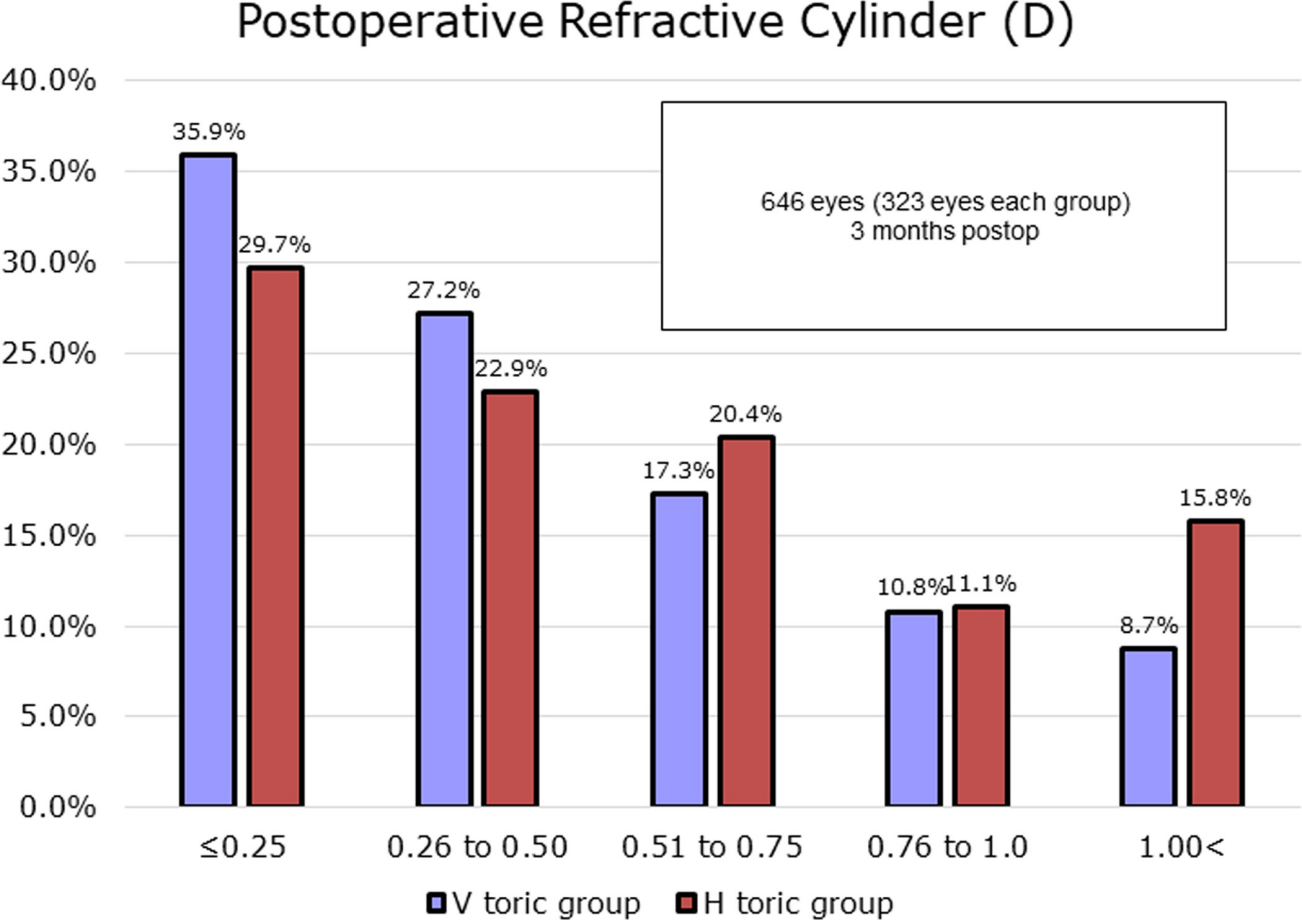
Refractive cylinder outcomes of vertical and horizontal implantation groups measured at 3-months postoperatively. N = 323 in each group.

Sixteen (5.0%) and 24 (7.4%) eyes required repositioning because the patients complained of visual acuity deterioration due to ICL rotation in the V and H toric groups, respectively, with no statistically significant difference (P = 0.146). Eight (2.5%) and 11 (3.4%) eyes were re-rotated after repositioning in the V and H toric groups, respectively, with no statistically significant difference (P = 0.485). Three (0.9%) and eight (2.5%) eyes required lens removal or replacement due to rotation in the V and H toric groups, respectively, with a statistically significant difference (P = 0.031; Table 3)

(Fig 3) shows the postoperative SE results for both groups at 3 months postoperatively. In the V toric group, 91.3% of eyes achieved postoperative SE within 0.5 D, while 2.2% of eyes had SE > 1.0 D. In the H toric group, 81.4% had postoperative SE within 0.5 D and 1.5% of eyes showed SE > 1.0 D.

(Fig 4) demonstrates the refractive cylinder outcomes of each group at 3 months postoperatively. The percentage of eyes with astigmatism within 0.25 D was 35.9% and 29.7% in the V and H toric groups, respectively. The proportion of astigmatism > 1.0 D was 8.7% and 15.8% in the V and H toric groups, respectively.

## Discussion

The number of patients with myopia has been increasing, as has the number of patients with high myopia who achieve insufficient results with corneal ablation [11]. ICL implantation has long been recognized as a good surgical method that eliminates the need for glasses in such patients, owing to its effectiveness and safety [12–14].

However, owing to the lens location in the sulcus and the absence of a specific fixing device, vision loss and patient dissatisfaction due to rotation after astigmatic ICL insertion have always been concerning to surgeons [15–17]. To maintain rotational stability, a slightly larger ICL size is recommended to ensure that it fits tightly within the sulcus; otherwise, serious irreversible problems such as increased IOP, anterior chamber angle closure, or a reduction of endothelial cells may occur [18].

To promote rotational stability, a vertical insertion method can be used [19]. The vertical distance is known to be longer than the horizontal distance, unlike the WTW distance [7, 20]. Specifically, if looking at the sulcus of the eye on a plane, an elongated oval shape is visible from top to bottom. When inserting a rectangular ICL vertically instead of horizontally, lens rotation can be prevented to a certain extent.

Rectangular ICLs are routinely inserted horizontally to prevent blocking the incised iris hole because a laser iridotomy is typically made on the upper iris when inserting an ICL without a hole. Even after developing an ICL with a central hole, the lens was still manufactured for the horizontal implantation method.

Since 2019, the direction of astigmatism has changed by 90° at our hospital. Rotational stability can be maintained with relatively low vaulting using a specially manufactured lens. A previous study reported that horizontal astigmatism lens implantation showed a postoperative rotation of approximately 3° in 52 eyes [21]. Another study compared horizontal with vertical ICL in 78 eyes of 46 patients and reported rotation of 3.27° and 1.74°, respectively, at 3 months postoperatively [19].

In our study, cases were selected by matching with a larger number of cases and the two groups were compared. The mean rotation degree was 1.11° and 3.02° in the V and H toric groups, respectively, showing similar results. Although slight differences may exist depending on the rotation measurement method, the horizontal ICL showed rotational stability of approximately 3°, whereas vertical ICL insertion showed rotational stability of approximately 1°.

The difference in rotational stability eventually appeared as a difference in MR astigmatism after surgery in the present study. No statistically significant difference was observed in the amount of astigmatism between groups in terms of absolute values, although the proportion of postoperative astigmatism within 0.5 D was higher in the V toric group, whereas cases with astigmatism exceeding 1.0 D were more common in the H toric group (Fig 4).

The correction effect in patients with astigmatism is known to diminish by approximately 3% per degree of rotation [22, 23]. Rotation of as little as 3° may not cause significant visual impairment. However, lens rotation > 5–7° usually causes a decrease in the patient’s visual acuity and discomfort; therefore, repositioning is often required [24, 25].

The differences were more pronounced for severe rotations requiring correction, removal, or lens replacement than those observed for small degrees of rotation. Rather, rotation of more than 30° toric lenses can cause new astigmatism [23]. Among the selected cases, none had complete rotation (> 30°) with vertical lens insertion. However, in cases of horizontal lens insertion, complete rotation was observed in 2.2%. In general, if the lens rotates excessively, removal of the lens is often necessary. Indeed, many eyes that underwent horizontal toric ICL implantation ultimately required a lens removal or a replacement. In cases of vertical toric insertion, a rectangular lens is inserted into a long ellipse superiorly and inferiorly, preventing further rotation even in cases of a rotation. However, in cases of horizontal lens insertion, it may rotate completely until it reaches the long axis, with a strong possibility that the lens will rotate again even after repositioning and require replacement due to severe visual acuity impairment (Fig 1).

The possibility of maintaining relatively low vaulting is another advantage of vertical lens insertion. In general, vaulting of approximately 500 μm is ideal [4]. If the vaulting is too high, complications such as endothelial cell loss and anterior angle obstruction may occur; if the vaulting is too low, the probability of cataracts increases [18]. However, in our experience, complications related to high vaulting are considered irreversible and relatively more dangerous than cataracts caused by low vaulting. In addition, cataract complications were reportedly further reduced in cases with low vaulting with the insertion of a V4c ICL (STAAR Surgical) capable of aqueous circulation facilitated by the hole [14]. In addition, when replacing the lens, it is more difficult to remove the larger lens than the smaller lens. Therefore, we have strived for low vaulting of < 500 μm for some time and have preferred horizontal toric ICL less often because the lower the vaulting with insertion, the greater the possibility of rotation [26]. However, rotational stability was achieved through vertical ICL insertion with relatively low vaulting, resulting in an explosive expansion of indications for toric lens implantation. Although not statistically significant, our findings revealed that the vaulting was slightly lower in the V toric group. Previous studies have also reported that the vaulting can be reduced by turning it vertically if it is too high after horizontal ICL insertion [8].

The main limitation of this study is that it was inevitably conducted retrospectively to secure a large number of cases. The first report of a prospective analysis of the rotational stability of a vertically inserted ICL was published during our study. Although only a few cases were included, this study confirmed that inserting a vertical ICL promotes rotational stability [19]. Therefore, our study provides more reliable results than previous studies by including large-scale clinical data, and our findings may be more impactful because the cases were carefully selected using strict 1:1 case matching. In particular, it has the advantage of strictly controlling astigmatism, ACD, STS, and ICL size, which can affect rotation and vaulting.

In conclusion, we evaluated the clinical results of vertical toric ICL implantation using large-scale case matching. In vertical toric ICL implantation cases, the rotational stability was superior to that of horizontal toric ICL implantation; fewer cases required lens replacement due to rotation, and stable visual acuity and astigmatism correction effects were observed. Vertical lens implantation is considered a good surgical method that can safely correct myopia and astigmatism in more patients with relatively low vaulting.

## Supporting information

S1 File STROBE statement—checklist of items that should be included in reports of observational studies. (DOCX)

## Abbreviations

ICL: implantable collamer lens
WTW: white-to-white
STS: sulcus-to-sulcus
ACD: anterior chamber depth
MR: manifest refraction
IOP: intraocular pressure
LogMAR CDVA: logarithm of the minimum angle of resolution corrected distance visual acuity
