# Supplementary material for: Vertical implantable collamer lens as a novel method to increase rotational stability
Source: PLoS One. 2024 Aug 19;19(8):e0308830. doi: 10.1371/journal.pone.0308830 (PMC11333007; doi:10.1371/journal.pone.0308830)
Supplement: S1 File — (DOCX) [file pone.0308830.s001.docx]

STROBE Statement—checklist of items that should be included in reports of observational studies

|  | Item No. | Recommendation | Page  No. | Relevant text from manuscript |
| --- | --- | --- | --- | --- |
| **Title and abstract** | 1 | (*a*) Indicate the study’s design with a commonly used term in the title or the abstract | 2 | **Design:** Retrospective comparative study |
|  |  | (*b*) Provide in the abstract an informative and balanced summary of what was done and what was found | 2 | Toric ICL vertical implantation showed good rotational stability, and appropriate visual acuity correction results with relatively low vaulting. This procedure therefore presents an effective novel method that could replace horizontal toric ICL implantation. |
| Introduction | | | |  |
| Background/rationale | 2 | Explain the scientific background and rationale for the investigation being reported | 3 | However, when relatively high vaulting occurs in clinical practice, endothelial cell reduction, iris chafing or peripheral anterior synechiae cause irreversible damage; therefore, the lens size should be considered to ensure the lowest possible vaulting. The ICL is generally inserted horizontally while considering the distance between the sulcus, based on the white-to-white (WTW) distance, as the manufacturer recommends. Then, if the vaulting is higher than expected, surgery must be performed to replace the lens. |
| Objectives | 3 | State specific objectives, including any prespecified hypotheses | 4 | Therefore, we aimed to perform a comparative study of the clinical results and rotation of hundreds of vertical ICL cases with those of horizontal toric ICL cases |
| Methods | | | |  |
| Study design | 4 | Present key elements of study design early in the paper | 4 | This study was conducted in accordance with the Declaration of Helsinki and was approved by the Institutional Review Board of Kangwon National University Hospital. We retrospectively analyzed the medical records of patients who underwent ICL implantation at Seoul Balgeunsesang Eye Clinic from 2003–2022. Rotation was compared using 1:1 matching by astigmatism (within 0.25 D), sulcus-to-sulcus (STS) distance (within 0.1 mm), anterior chamber depth (ACD) (within 0.1 mm), and ICL size (12.1, 12.6, 13.2, or 13.7 mm) between the eyes that received horizontal toric ICL (H toric group) and those that received vertical toric ICL (V toric group). |
| Setting | 5 | Describe the setting, locations, and relevant dates, including periods of recruitment, exposure, follow-up, and data collection | 4 | This study was conducted in accordance with the Declaration of Helsinki and was approved by the Institutional Review Board of Kangwon National University Hospital. We retrospectively analyzed the medical records of patients who underwent ICL implantation at Seoul Balgeunsesang Eye Clinic from 2003–2022. Rotation was compared using 1:1 matching by astigmatism (within 0.25 D), sulcus-to-sulcus (STS) distance (within 0.1 mm), anterior chamber depth (ACD) (within 0.1 mm), and ICL size (12.1, 12.6, 13.2, or 13.7 mm) between the eyes that received horizontal toric ICL (H toric group) and those that received vertical toric ICL (V toric group). |
| Participants | 6 | (*a*) *Cohort study*—Give the eligibility criteria, and the sources and methods of selection of participants. Describe methods of follow-up  *Case-control study*—Give the eligibility criteria, and the sources and methods of case ascertainment and control selection. Give the rationale for the choice of cases and controls  *Cross-sectional study*—Give the eligibility criteria, and the sources and methods of selection of participants | 4 | We retrospectively analyzed the medical records of patients who underwent ICL implantation at Seoul Balgeunsesang Eye Clinic from 2003–2022. |
|  |  | (*b*) *Cohort study*—For matched studies, give matching criteria and number of exposed and unexposed  *Case-control study*—For matched studies, give matching criteria and the number of controls per case | 4 | Rotation was compared using 1:1 matching by astigmatism (within 0.25 D), sulcus-to-sulcus (STS) distance (within 0.1 mm), anterior chamber depth (ACD) (within 0.1 mm), and ICL size (12.1, 12.6, 13.2, or 13.7 mm) between the eyes that received horizontal toric ICL (H toric group) and those that received vertical toric ICL (V toric group). |
| Variables | 7 | Clearly define all outcomes, exposures, predictors, potential confounders, and effect modifiers. Give diagnostic criteria, if applicable | 6 | Follow-up was performed on the first day, week, and month postoperatively, the third month postoperatively, and annually thereafter. Three months after surgery, the uncorrected and corrected visual acuity, MR, IOP, lens rotation by dilation, and vaulting were measured using anterior optical coherence tomography (Cirrus 6000, Carl Zeiss Meditec AG, Jena, Germany). To ensure accurate angle measurement, anterior segment images were obtained, and the rotation angle was measured by inserting the image into a smartphone protractor application |
| Data sources/ measurement | 8* | For each variable of interest, give sources of data and details of methods of assessment (measurement). Describe comparability of assessment methods if there is more than one group | 6 | Follow-up was performed on the first day, week, and month postoperatively, the third month postoperatively, and annually thereafter. Three months after surgery, the uncorrected and corrected visual acuity, MR, IOP, lens rotation by dilation, and vaulting were measured using anterior optical coherence tomography (Cirrus 6000, Carl Zeiss Meditec AG, Jena, Germany). To ensure accurate angle measurement, anterior segment images were obtained, and the rotation angle was measured by inserting the image into a smartphone protractor application |
| Bias | 9 | Describe any efforts to address potential sources of bias | 6 | Rotation was compared using 1:1 matching by astigmatism (within 0.25 D), sulcus-to-sulcus (STS) distance (within 0.1 mm), anterior chamber depth (ACD) (within 0.1 mm), and ICL size (12.1, 12.6, 13.2, or 13.7 mm) between the eyes that received horizontal toric ICL (H toric group) and those that received vertical toric ICL (V toric group). |
| Study size | 10 | Explain how the study size was arrived at | 6 | Of the 16,281 eyes, 1,850 had lenses inserted vertically (the rest, 14,431, had been inserted using the prior horizontal method). Six hundred forty-six eyes from 646 individuals were selected according to the matching criteria (323 eyes each in the V and H toric groups, respectively). |

Continued on next page

| Quantitative variables | 11 | Explain how quantitative variables were handled in the analyses. If applicable, describe which groupings were chosen and why | 6 | The size of the study subjects was calculated using the g-power program (ver. 3.1.9.7, Heinrich-Heine-Universität Düsseldorf, Düsseldorf, Germany). For a power of 0.99, 296 cases were required (effect size 0.5, alpha-error: 0.05), and more than 300 cases were collected through a 1:1 match.  Statistical analysis was performed using SPSS version 25.0 (IBM, Armonk, NY, USA). We used the Kolmogorov-Smirnov test to verify the normal distribution of the data. An independent t-test was performed to compare numeric variables; the chi-square test was performed to compare categorical variables. P values less than 0.05 were considered statistically significant. To ensure statistical accuracy. Only the right eye was included in the statistical analysis when both eyes met the criteria. |
| --- | --- | --- | --- | --- |
| Statistical methods | 12 | (*a*) Describe all statistical methods, including those used to control for confounding | 6 | The size of the study subjects was calculated using the g-power program (ver. 3.1.9.7, Heinrich-Heine-Universität Düsseldorf, Düsseldorf, Germany). For a power of 0.99, 296 cases were required (effect size 0.5, alpha-error: 0.05), and more than 300 cases were collected through a 1:1 match.  Statistical analysis was performed using SPSS version 25.0 (IBM, Armonk, NY, USA). We used the Kolmogorov-Smirnov test to verify the normal distribution of the data. An independent t-test was performed to compare numeric variables; the chi-square test was performed to compare categorical variables. P values less than 0.05 were considered statistically significant. To ensure statistical accuracy. Only the right eye was included in the statistical analysis when both eyes met the criteria. |
|  |  | (*b*) Describe any methods used to examine subgroups and interactions |  |  |
|  |  | (*c*) Explain how missing data were addressed |  |  |
|  |  | (*d*) *Cohort study*—If applicable, explain how loss to follow-up was addressed  *Case-control study*—If applicable, explain how matching of cases and controls was addressed  *Cross-sectional study*—If applicable, describe analytical methods taking account of sampling strategy |  |  |
|  |  | (*e*) Describe any sensitivity analyses | 6 | Statistical significance was set at *p* < 0.05. |
| Results | | | | |
| Participants | 13* | (a) Report numbers of individuals at each stage of study—eg numbers potentially eligible, examined for eligibility, confirmed eligible, included in the study, completing follow-up, and analysed | 6 | Of the 16,281 eyes, 1,850 had lenses inserted vertically (the rest, 14,431, had been inserted using the prior horizontal method) |
|  |  | (b) Give reasons for non-participation at each stage |  |  |
|  |  | (c) Consider use of a flow diagram |  |  |
| Descriptive data | 14* | (a) Give characteristics of study participants (eg demographic, clinical, social) and information on exposures and potential confounders | 7 | The average participant age was 24.70 ± 4.89 and 24.82 ± 4.67 years in the V and H toric groups, respectively (p = 0.742). The spherical equivalent (SE) was –8.60 ± 1.91 and –8.55 ± 1.89D in the V and H toric groups, respectively (p=0.770). Preoperative ophthalmologic measures such as logarithm of the minimum angle of resolution corrected distance visual acuity (logMAR CDVA), astigmatism, IOP, keratometry, WTW and STS distances, ACD, and corneal thickness showed no statistically significant differences between groups. |
|  |  | (b) Indicate number of participants with missing data for each variable of interest |  |  |
|  |  | (c) *Cohort study*—Summarise follow-up time (eg, average and total amount) |  |  |
| Outcome data | 15* | *Cohort study*—Report numbers of outcome events or summary measures over time |  |  |
|  |  | *Case-control study—*Report numbers in each exposure category, or summary measures of exposure |  |  |
|  |  | *Cross-sectional study—*Report numbers of outcome events or summary measures | *6* | Of the 16,281 eyes, 1,850 had lenses inserted vertically (the rest, 14,431, had been inserted using the prior horizontal method). Six hundred forty-six eyes from 646 individuals were selected according to the matching criteria (323 eyes each in the V and H toric groups, respectively) |
| Main results | 16 | (*a*) Give unadjusted estimates and, if applicable, confounder-adjusted estimates and their precision (eg, 95% confidence interval). Make clear which confounders were adjusted for and why they were included | 8 | Overall, 147 eyes (45.5%) in the V toric group showed no postoperative lens rotation, which was statistically significantly higher compared to 101 eyes in the H toric group (31.3%) with no lens rotation (P <0.001) |
|  |  | (*b*) Report category boundaries when continuous variables were categorized |  |  |
|  |  | (*c*) If relevant, consider translating estimates of relative risk into absolute risk for a meaningful time period |  |  |

Continued on next page

| Other analyses | 17 | Report other analyses done—eg analyses of subgroups and interactions, and sensitivity analyses |  |  |
| --- | --- | --- | --- | --- |
| Discussion | | | | |
| Key results | 18 | Summarise key results with reference to study objectives | 11 | In our study, cases were selected by matching with a larger number of cases and the two groups were compared. The mean rotation degree was 1.11° and 3.02° in the V and H toric groups, respectively, showing similar results. Although slight differences may exist depending on the rotation measurement method, the horizontal ICL showed rotational stability of approximately 3°, whereas vertical ICL insertion showed rotational stability of approximately 1 |
| Limitations | 19 | Discuss limitations of the study, taking into account sources of potential bias or imprecision. Discuss both direction and magnitude of any potential bias | 12 | The main limitation of this study is that it was inevitably conducted retrospectively to secure a large number of cases. The first report of a prospective analysis of the rotational stability of a vertically inserted ICL was published during our study. Although only a few cases were included, this study confirmed that inserting a vertical ICL promotes rotational stability. [18] Therefore, our study provides more reliable results than previous studies by including large-scale clinical data, and our findings may be more impactful because the cases were carefully selected using strict 1:1 case matching. In particular, it has the advantage of strictly controlling astigmatism, ACD, STS, and ICL size, which can affect rotation and vaulting. |
| Interpretation | 20 | Give a cautious overall interpretation of results considering objectives, limitations, multiplicity of analyses, results from similar studies, and other relevant evidence | 12 | The possibility of maintaining relatively low vaulting is another advantage of vertical lens insertion. In general, vaulting of approximately 500 µm is ideal. [4] If the vaulting is too high, complications such as endothelial cell loss and anterior angle obstruction may occur; if the vaulting is too low, the probability of cataracts increases. [17] However, in our experience, complications related to high vaulting are considered irreversible and relatively more dangerous than cataracts caused by low vaulting. In addition, cataract complications were reportedly further reduced in cases with low vaulting with the insertion of a V4c ICL (STAAR Surgical) capable of aqueous circulation facilitated by the hole. [13] In addition, when replacing the lens, it is more difficult to remove the larger lens than the smaller lens. Therefore, we have strived for low vaulting of < 500 μm for some time and have preferred horizontal toric ICL less often because the lower the vaulting with insertion, the greater the possibility of rotation. [25] However, rotational stability was achieved through vertical ICL insertion with relatively low vaulting, resulting in an explosive expansion of indications for toric lens implantation. Although not statistically significant, our findings revealed that the vaulting was slightly lower in the V toric group. Previous studies have also reported that the vaulting can be reduced by turning it vertically if it is too high after horizontal ICL insertion. |
| Generalisability | 21 | Discuss the generalisability (external validity) of the study results | 13 | In conclusion, we evaluated the clinical results of vertical toric ICL implantation using large-scale case matching. In vertical toric ICL implantation cases, the rotational stability was superior to that of horizontal toric ICL implantation; fewer cases required lens replacement due to rotation, and stable visual acuity and astigmatism correction effects were observed. Vertical lens implantation is considered a good surgical method that can safely correct myopia and astigmatism in more patients with relatively low vaulting. |
| Other information | |  | | |
| Funding | 22 | Give the source of funding and the role of the funders for the present study and, if applicable, for the original study on which the present article is based |  | N/A |

*Give information separately for cases and controls in case-control studies and, if applicable, for exposed and unexposed groups in cohort and cross-sectional studies.

**Note:** An Explanation and Elaboration article discusses each checklist item and gives methodological background and published examples of transparent reporting. The STROBE checklist is best used in conjunction with this article (freely available on the Web sites of PLoS Medicine at http://www.plosmedicine.org/, Annals of Internal Medicine at http://www.annals.org/, and Epidemiology at http://www.epidem.com/). Information on the STROBE Initiative is available at www.strobe-statement.org.
